# The Molecular Basis of Alcohol Use Disorder (AUD). Genetics, Epigenetics, and Nutrition in AUD: An Amazing Triangle

**DOI:** 10.3390/ijms22084262

**Published:** 2021-04-20

**Authors:** Agnieszka Siomek-Gorecka, Anna Dlugosz, Damian Czarnecki

**Affiliations:** 1 Department of Clinical Biochemistry, Faculty of Pharmacy, Collegium Medicum in Bydgoszcz, Nicolaus Copernicus University in Toruń, 85-095 Bydgoszcz, Poland; 2 Department of Engineering and Chemical and Food Analytics, Faculty of Chemical Technology and Engineering, UTP University of Science and Technology, 85-326 Bydgoszcz, Poland; anna.dlugosz@utp.edu.pl; 3 Department of Preventive Nursing, Faculty of Health Sciences, L. Rydygier Collegium Medicum in Bydgoszcz, Nicolaus Copernicus University in Toruń, 85-821 Bydgoszcz, Poland; czarneckidamian@cm.umk.pl

**Keywords:** epigenetics, genetics, nutrition, alcohol use disorder

## Abstract

Alcohol use disorder (AUD) is a very common and complex disease, as alcohol is the most widely used addictive drug in the world. This disorder has an enormous impact on public health and social and private life, and it generates a huge number of social costs. Alcohol use stimulates hypothalamic–pituitary–adrenal (HPA) axis responses and is the cause of many physical and social problems (especially liver disease and cancer), accidental injury, and risky sexual behavior. For years, researchers have been trying to identify the genetic basis of alcohol use disorder, the molecular mechanisms responsible for its development, and an effective form of therapy. Genetic and environmental factors are known to contribute to the development of AUD, and the expression of genes is a complicated process that depends on epigenetic modulations. Dietary nutrients, such as vitamins, may serve as one these modulators, as they have a direct impact on epigenomes. In this review, we connect gathered knowledge from three emerging fields—genetics, epigenetics, and nutrition—to form an amazing triangle relating to alcohol use disorder.

## 1. Introduction

**Figure 1:**
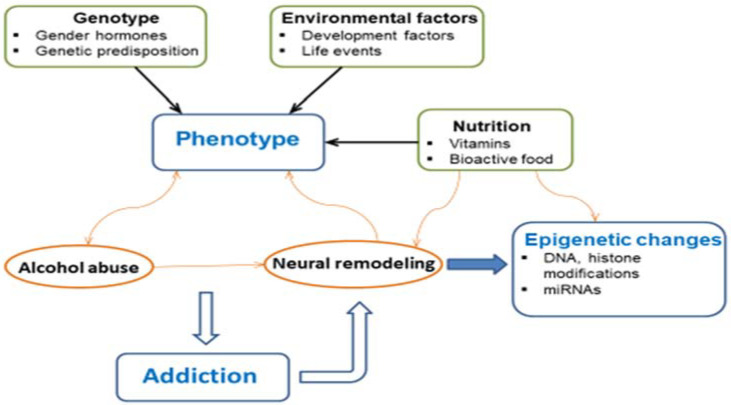
Mutual associations among nutrition, genetic background with epigenetic changes, and environmental factors forming the basis of addiction (Modified from Beayno, et al. [2]). The expression of a phenotype, both on a cellular and organismal level, is not only dependent on the hereditary luggage but may be also modulated by nutritional and environmental factors. This is the interplay of nonmodifiable variables describing the genotype and modifiable variables describing nutritional and environmental factors. Alcohol abuse modifies the structure of chromatin and modulates gene expression through epigenetic changes. In a feedback loop, this neural remodeling conversely reinforces the abuse of alcohol. This is hypothesized to move alcohol abuse through the stages that eventually lead to addiction.

Alcohol use disorder (AUD) is a complex, multifaceted psychiatric condition. Its development and regulation are believed to be products of both genetic and environmental influences on the human brain. These factors may also increase susceptibility to the development of alcohol addiction. Recent research on alcohol exposure suggests that genome chemical modifications, called epigenetic mechanisms, are important molecular factors that may be helpful in the discovery of the pathogenesis of AUD. Alcohol addiction is also genetically complex and includes genetic heterogeneity at the level of neurobiological vulnerability, polygenicity, phenocopies, and gene and environment interaction [1]. As shown in Figure 1, mutual associations among the abovementioned factors seem to play an important role in the development of addictions.

In the selection of articles, we were directed by our areas of specialization, our clinical work, and the aim of our future study, which is interdisciplinary, including epigenetic DNA modifications, dietetics, and addiction. Our articles are most frequently searched for in the National Center for Biotechnology Information (PubMed.gov, accessed on 10 February 2021).

## 2. Alcohol Metabolism and Genetic Polymorphisms in Alcohol Addiction

The identification of the genes that predispose individuals to alcohol addiction is very important. The products of these genes are responsible for the human answer to alcohol exposure (ethanol metabolizing enzymes) and clinical treatment, which may modulate the interaction with environmental factors. The process of addiction involves a cellular molecule network, in which hundreds of genes play very important roles. Products of these genes act as neurobiological factors in processes like rewards, behavioral control, and stress reactions. While they are also an important part of the development of mental diseases, a group of genes strictly involved in alcohol metabolism has also been detected, and their polymorphisms may lead to serious health consequences [3].

### 2.1. Genes and Enzymes Involved in Alcohol Metabolism

Alcohol is metabolized in the human body by various mechanisms. The oxidative metabolism pathway needs two main enzymes, including alcohol dehydrogenase (ADH), which oxidizes ethanol to the highly toxic acetaldehyde. Then, the aldehyde dehydrogenase (ALDH) converts this into the nontoxic acetate and eventually into acetyl CoA. Acetyl CoA is metabolized to the water and carbon dioxide for easy elimination [4]. Through this process, depending on the nutritional, hormonal, and energetic conditions, the acetyl CoA may be converted into CO_2_, ketone bodies, fatty acid, and cholesterol. Apart from these two very important enzymes, ethanol is metabolized by others, such as cytochrome P4502E1 (CYP2E1) and catalase, which also take part in the production of acetaldehyde from ethanol oxidation. The microsomal ethanol-oxidizing system mostly involves the cytochrome CYP2E1 and two others—CYP1A2 and CYP3A4. Microsomal ethanol oxidization represents the main non-ADH ethanol metabolism liver system [5]. The alternative ethanol metabolism pathway is nonoxidative. The first pathway reaction is catalyzed by the enzyme fatty acid ethyl ester (FAEE) synthase and leads to the formation of FAEEs. Fatty acid ethyl esters (FAEEs) have been implicated as mediators of ethanol-induced organ damage. It has been shown that FAEE synthase is present selectively in the organs commonly damaged by ethanol abuse [6]. The second involves the formation of a phospholipid, known as a phosphatidyl ethanol (PEth), which has become a specific and sensitive alcohol biomarker [7].

#### 2.1.1. The Alcohol Dehydrogenase 1B (ADH1B) and Aldehyde Dehydrogenase 2 (ALDH2) Genes

A convincing example of the role of genes in alcohol use disorder are two genes mentioned above, the products of which are strongly linked to alcohol metabolism: the alcohol dehydrogenase 1B (ADH1B) and aldehyde dehydrogenase 2 (ALDH2) genes. In humans, on the basis of structural properties and kinetics, the enzyme ADH has been categorized into five classes, but the main isoforms involved in ethanol metabolism are ADHs from I, II, and IV classes. Two of the three ADH1 enzymes, ADH1B and ADH1C, show genetic polymorphisms (Table 1). Genetic polymorphisms in the ADH1B and ADH1C gene locations are associated with a different enzyme activity [8].

Among the many isozymes of ALDH, only the cytosolic ALDH1 and mitochondrial ALDH2 can metabolize acetaldehyde (Table 2). A significant role in acetaldehyde oxidation has been observed only for ALDH1A1, ALDH1B1, and ALDH2 isoforms. However, mitochondrial ALDH2 plays a central role in human acetaldehyde metabolism [3]. Genetic polymorphisms of ADH and ALDH have been shown to be linked with alcohol consumption habits, as well as susceptibility to the development of alcohol abuse and alcohol addiction. Two variations of the ALDH1 enzyme, ALDH1A1*2 and ALDH1A1*3, may be associated with alcohol addiction in Afro-Americans [10].

Special attention was paid to the genetic variants of both the *ADH1B* and *ADH1C* genes. The *ADH1B2, ADH1B3*, and *ADH1C1* variants have a faster rate of ethanol oxidation, so they lead to acetaldehyde accumulation. ADH1B*1 is the predominant allele in all populations. In some Asian populations, ADH1B*2 may be found in 90% of the population. In Caucasian populations, ADH1C*1 and ADH1C*2 appear with equal frequency [12], whereas the ADH1C*1 allele is present in about 50% of Europeans. Allele ADH1B*3 occurs mainly in Africans, African-Americans, and some native Americans [13]. The ADH1B*2 allele has been shown to decrease the occurrence of alcohol abuse and alcohol addiction in Asians and in the Caucasian and Jewish populations [14]. Interestingly, several studies have reported that *ADH1B* allele frequencies differ between alcoholics and non-alcoholics, with a higher occurrence of the atypical form, ADH1B*2, in non-alcoholics [15]. Meta-analyses have suggested that the ADH1B*1 allele is associated with a three-fold increase in the risk of alcoholism relative to the ADH1B*2 [16], which is considered as a protective function against alcohol abuse and alcoholism. While this protection effect was believed to cause unpleasant symptoms connected with acetaldehyde accumulation after alcohol consumption [17], the frequency of facial flushing (a typical result of acetaldehyde accumulator) was shown to be similar in individuals carrying different *ADH1B* alleles [18]. Additionally, the primary flushing effect is believed to be a result of the higher *ADH1B* activity and lower ALDH2 activity and was also found to be independent of the ADH1B*2 and ALDH2*2 alleles [19]. Some reports have underlined that the most significant functional gene loci are the His47Arg polymorphisms in the *ADH1B* gene, where Arg47 is the overactive version, and the ALDH2 Glu487Lys, in which the Lys487 allele deactivates *ALDH2* [1]. In the Chinese and Japanese as well as in Jewish populations, when both His47 and Lys487 are plentiful, some people carry genotypes that protect them from the development of alcohol addiction. Finally, the precise mechanism of gene polymorphism associated with alcoholism susceptibility or resistance needs to be clarified.

#### 2.1.2. The Microsomal Ethanol Oxidizing System

This system mostly involves CYP2E1 and also the cytochromes CYP1A2 and CYP3A4. CYP2E1 is active only after the consumption of a large amount of alcohol. After chronic ethanol consumption, the activity of the microsomal ethanol-oxidizing system (MEOS) increases, with an associated rise in P-450 cytochromes, especially CYP2E1. When alcohol is metabolized by CYP2E1, highly reactive oxygen species (ROS) are produced. According to many discrepancies among studies concerning the association between CYP2E1 functional polymorphism and alcohol addiction, this polymorphism is not believed to be related to alcohol abuse [3]. Genetic catalase polymorphism also still needs further investigation. As has been shown, a common polymorphism in the promoter region of the catalase gene, CAT c.-262C > T, has an impact on alcohol dependence and its severity [20]. It was found that CAT levels were significantly higher in subjects carrying the CAT0262 t allele [20]. Interestingly, some studies have observed that blood catalase activity significantly correlates with alcohol consumption, and human brain catalase activity modulates alcohol consumption urges [21]. The role of catalase in alcohol use disorder is supported by the results of a study showing that subjects with a family history of alcoholism have a higher catalase activity than a control group [22].

Due to the aforementioned information, pharmaceutical manipulation of ethanol metabolism in humans is based on ALDH inhibition. ALDH inhibitors, such as disulfiram (Antabuse) and calcium carbimide (Abstem, Temposil), are used as the basis for treating chronic alcoholics. Due to the limited efficacy of current medications, personalized treatment is required in the clinical management of patients. The next medicine, the mechanism of which is strictly related to the genotype of patients, is naltrexone. Naltrexone is a μ-opioid receptor antagonist that is also used in the treatment of alcoholics with a moderate efficacy, and the gene encoding the μ-opioid receptor1 (*OPRM1*) is becoming the focus of studies, describing a functional polymorphism within the coding sequence of this gene (Asn40Asp) [23]. Moreover, in this case, there are conflicting study data, although many seem promising, showing that the functionally significant *OPRM1* Asp40 allele predicts naltrexone treatment response in alcoholic individuals, so that *OPRM1* genotyping in alcoholic individuals might be useful for the selection of treatment options [24]. On the other hand, the response to alcohol consumption may be different due to changes in opioid receptors.

### 2.2. Gene and Environmental Interactions

The development of alcohol addiction is a very complex process, in which the interaction between genes and the environment plays a crucial role. Environmental factors include the availability of alcohol, parental attitudes, childhood maltreatment, and peer pressure. Gene and environment interactions (GxE) occur when the effect of exposure to an environmental factor on a person’s health is conditioned by the genotype. The gene–environment effects within psychiatric disorders have been described for many genes, such as monoamine oxidase A (*MAOA*), the serotonin transporter (*HTT*), *COMT*, the corticotrophin-releasing hormone receptor 1 gene, and the dopamine transporter [25,26,27].

#### 2.2.1. The Catechol-O-Methyltransferase Gene (COMT)

One of the genes that are important in the development of AUD is catechol-O-methyltransferase (COMT). This enzyme metabolizes dopamine, norepinephrine, and other catecholamines. Val158Met is a common functional polymorphism, located in the human *COMT* gene coding sequence, in which the amino acid at codon 158 may be either valine (Val) or methionine (Met). The Val158 allele is three to four times more active than Met158, and the alleles act co-dominantly [28,29]. There are no clear data on these two alleles’ effects in alcohol addiction, as it was found in certain addicted populations (e.g., polysubstance abusers) that Val158 is associated with addiction, whereas in others, such as late-onset alcoholics in Finland and Finnish social drinkers, increased risk seemed to be connected with the Met158 allele [30,31]. Studies have confirmed that the changes in the dopaminergic system due to polymorphisms of the DRD2 and DRD4 genes are connected with a higher risk of alcohol consumption [32]. Many studies have also shown that different variants of gene polymorphisms of receptor DRD2 rs1799732 and DRD4 VNTR determine temperament traits, such as impulsivity, which is conducive to the deterioration of self-control (e.g., difficulty controlling alcohol consumption and abstinence) [33].

#### 2.2.2. The Monoamine Oxidase A Gene (MAOA)

*MAOA* is an X-linked gene-encoding monoamine oxidase A, a mitochondrial enzyme that metabolizes monoamine neurotransmitters, including norepinephrine, dopamine, and serotonin. A well-known polymorphism, called the *MAOA*-linked polymorphic region (*MAOA-LPR*), is a variable-number tandem repetition (VNTR), which consists of a number of different copies of a 30 base pair (bp) repeated sequence, with three and four repeat alleles being by far the most common. Alleles with four repeats are transcribed more efficiently than three repeat copies and are associated with a higher MAOA activity [34]. It has been shown that in women, the effect of childhood sexual abuse on the risk of developing alcohol addiction was connected with the *MAOA-LPR* genotype. Sexually abused women who were homozygous for the low-activity *MAOA-LPR* allele had higher rates of problem drinking, especially antisocial alcohol use, compared with those who were homozygous for the high-activity allele [35].

#### 2.2.3. The Serotonin Transporter Gene (HTT)

*HTT* is responsible for serotonin re-uptake and works as a crucial regulator of serotonin availability in the synaptic cleft. A common polymorphism of the HTT promoter region (*5-HTTLPR*) affects expression, with the major alleles involving 16 (L) or 14 (S) copies of a 20–30 bp imperfectly repeated sequence [36]. It has also been shown that *5-HTTLPR* is actually a functionally tri-allelic locus due to functional A > G substitution within the L allele. While the s allele, with a low transcription rate, has been related to anxiety and alcohol addiction, the effect of this allele on behavior appears to be stronger when there is stress exposure. *5-HTTLPR* has been shown to reassure some brain functions in parts that are critical for emotional regulation and response to environmental changes and may moderate the impact of stressful life events on the risk of depression and suicide [37,38]. Additionally, macaque orthologous *rs-5HTTLPR* polymorphism was observed to influence alcohol consumption and stress response, depending on the conditions of upbringing. Carriers of the genotype, with a low expression, who had been separated from their mother at an early age displayed a higher stress reactivity and ethanol preference [39].

### 2.3. Oxidative Stress in Alcohol Use Disorder

Reactive oxygen species (ROS) are generated during ethanol metabolism. ROS are highly reactive and capable of damaging various molecules, including proteins, lipids, carbohydrates, and DNA. The status of biochemical parameters and antioxidants were measured in 28 patients with alcohol dependence in a clinical study in Taiwan [40]. Malondialdehyde (MDA) is a marker of lipid oxidation, and its increased level was found in a group of patients. Furthermore, the duration of alcohol dependence was significantly correlated with MDA levels. The superoxide dismutase (SOD) and glutathione peroxidase (GPX) activity were lower in that group, which emphasized the impairment of antioxidant defense and oxidative stress occurrence [40]. While oxidative damage to most molecules may be easily repaired, oxidative DNA damage may lead to serious biological changes. Among the various forms of oxidative DNA damage is a reaction with the C-8 position of the guanine base on DNA, resulting in the generation of 8 oxo-guanine (8-oxoGua) and its nucleoside 8-oxo-7,8-dihydro-2′-deoxyguanosine (8-oxodG) or 8-hydroxy-2′deoxyguanosine (8-OHdG), the most widely studied and best recognized marker of oxidatively modified DNA [41,42]. Oxidative DNA damage, measured as a level of 8-hydroxy-2′deoxyguanosine (8-OHdG), was shown to be higher in alcohol-dependent patients (79 persons) than in a control group (63 healthy persons). In addition, authors have observed that this damage persisted after 1 week of detoxification, and alcohol withdrawal syndrome (AWS) was correlated with the level of oxidative DNA damage [43]. The authors have concluded that there are some limitations of their results, such as the short period of abstinence, the dependence of oxidative DNA damage on many factors, and the observation of an increased level of this damage in other central disorders. However, their study has certainly shown that alcohol-dependent persons are susceptible to an excessive production of free radicals and, consequently, its harmful effects. The study of animal models has shown that a high concentration of ethanol connected with a vitamin-depleted diet increased the level of 8-oxoGua and its repair activity in the liver and esophagus, which may be a risk factor in the development of cancer. However, authors have found that in animals treated with carcinogen, a lower level of ethanol decreased 8-oxoGua and its repair activity in the analyzed organs. On the basis of the experiments conducted, the authors concluded that the effect of ethanol consumption on cancer risk, including the generation of 8-oxoGua, depends on the ethanol concentration and diet [44]. Another study on ethanol-fed pigs and a control group has revealed that the effect of ethanol on oxidative stress intensification is not obvious, as no significant differences in the 8-oxodG and MDA levels between analyzed groups were found [45]. This small experiment conducted on pigs (*n* = 4 in every group) has shown that alcohol consumption for 39 days may not cause oxidative damage to DNA and lipids. According to the authors, the critical determinants of ethanol toxicity may be the duration of alcohol uptake and alcohol-induced nutritional deficiency. Some authors have indicated that chronic ethanol exposure up-regulates the production of ROS and NO in human neurons, and chronic oxidative stress initiates neuronal injury [46]. In addition, they have observed that both alcohol-metabolizing enzymes (ADH and CYP2E1) are active in human neurons, and their activities are higher after EtOH exposure. Our study has shown that the level of 8-oxoguanine in cerebrospinal fluid and urinary excretion of oxidative DNA damage repair products were higher in mixed Alzheimer disease/vascular dementia (MD) patients than in a control group [47], which supported the observation that oxidative stress is one of the mechanisms leading to neural dysfunction. While oxidative stress and oxidative DNA damage have been implicated in the progression of many neurodegenerative disorders, and numerous studies have reported that a large number of detoxified alcoholics have cognitive or memory disturbances [48], one cannot exclude a possible link to the loss of neural plasticity and other changes observed in the brain of an alcoholic. All this information underlines the role of oxidative stress/oxidative DNA damage in alcohol-dependent disorders, especially in the context of cancer development and alcohol-induced oxidative stress in the central nervous system.

## 3. Epigenetics

Alcohol addiction is a chronic, relapsing brain disorder, which is characterized by a compulsion to seek alcohol, loss of control in limiting alcohol intake, and negative emotional state during withdrawal [49], in which genetic and environmental factors interact and appear to be equally important with respect to its development [50]. Recent studies have shown that every cell under the influence of environmental stressors may express a new phenotype without genetic changes. This also takes place in several nervous system nuclei. Thus, in addition to environmental stressors, epigenetic modifications can lead to chronic changes in gene expression and, as a consequence, to vulnerability to addiction [2]. Moreover, it was observed that both stress and addiction can induce similar epigenetic modifications and underlying changes in neurochemical pathways and synaptic plasticity, which suggests a link between alcohol use disorder and stress-related disorders [51].

The term epigenetics refers to the chemical modifications occurring within a genome that may modulate gene expression, without changing the DNA sequence [52]. It is common knowledge that epigenetic mechanisms play a crucial role in regulating gene expression, as these mechanisms can transiently or stably manipulate this expression. Their main pathways involve DNA methylation and covalent modifications of histones, which may undergo methylation, acetylation, phosphorylation, or ubiquitination reactions. In the case of DNA modification, the main enzymes are methyltransferases (DNTMs), which relocate a methyl group from S-adenosyl-methionine (SAM) to the target cytosine. These DNMTs are abundant in fully differentiated adult neurons and are believed to play a crucial role in the regulation of gene expression [53]. Histones may be altered through a plethora of enzymes, such as histone acetyltransferases (HATs), histone deacetylases (HDACs), histone methyltransferases (HMTs), and histone demethylases (HDMs) [54]. Histones and DNA modifications can result in the remodeling of the structure of protein–DNA complexes, thereby regulating the access of transcriptional machinery to the DNA and, finally, cellular gene expression [55].

### 3.1. DNA Methylation

The best-known epigenetic DNA modification is the methylation of cytosine at the C5 position in CpG dinucleotides, located mostly in the promoter regions of DNA, with the creation of 5-metylcytosine (5-mCyt). More than 28 million CpG sites are distributed across the human genome, and 70–80% of them can be methylated [56]. Methylation is usually associated with gene transcription silencing and, in general, is believed to be a stable modification [55]. While for years, there was only certainty about native demethylation, recent data have shown that active demethylation processes exist and could be related to the pathogenesis of many diseases, such as cancer [57,58,59]. Accumulating evidence indicates that DNA methylation is reversible, especially in the brain, which may be crucial in relation to genes associated with addiction. In the active demethylation reaction cascade, one key element is the participation of the ten-eleven-translocation enzymes, TET 1-3. These enzymes mediate the conversion of 5-methylcytosine (5-mCyt) into 5-hydroxymethylcytosine (5-hmCyt) and perform further oxidation reactions that generate 5-formylcytosine (5-fCyt) and 5-carboxycytosine (5-caCyt) [60,61]. Then, the activated base excision repair (BER) pathway, using thymine DNA glycosylase (TDG), replaces this modification with cytosine. Thus, 5-mCyt oxidation is a plausible DNA demethylation mechanism [62]. Some studies have confirmed the observation that 5-hmCyt is most abundant in the brain, compared with the other organs [63], and emphasized its role in neural function [53]. Our study confirmed this observation, as we found a higher level of 5-mdC and 5-hmC in mice brain tissue in comparison with the kidney and liver [64]. A person’s susceptibility to alcoholism-related brain damage may be associated with his or her age, gender, drinking history, and nutrition, as well as with the vulnerability of specific brain regions [65]. The intermediate products of the DNA demethylation pathway have been analyzed in our laboratory using online automated isotope-dilution two-dimensional ultra-performance liquid chromatography with tandem mass spectrometry (2D-UPLC MS/MS). In recent years, the results of our analyses have shown the strong impact of epigenetic modifications in diseases and human conditions, as well as in the NF-kappa B signaling pathway in Cu,Zn-SOD-deficient mice [64,66,67].

Studies of patients with alcoholism have shown a significant increase in genomic DNA methylation in the whole blood, which was associated with decreased DNMT-3a and DNMT-3b mRNA levels. This observation suggests a feedback regulation of these enzymes by the increased DNA methylation [68]. In addition, in this study, the authors observed a significant negative correlation between DNMT-3b expression and blood alcohol concentration. An increased DNA methylation in the medial pre-frontal cortex and decreased expression of proteins involved in synaptic neurotransmitter release were also shown in alcohol-dependent rats. Additionally, the authors have observed that the administration of a DNA methyltransferase inhibitor prevented increased drinking behavior post-abstinence, which suggests the therapeutic potential of DNA methyltransferases inhibitors [69]. DNA demethylases, DNMTa, DNMT3a, and 3b, are widely expressed in the nervous system. It has been shown in multiple studies that drugs modify the expression of DNMTs [70,71]. Other studies have indicated that MeCP2 (an epigenetic factor that binds methylated cytosine and act as a transcriptional repressor) mediates behavioral responses to alcohol and addictive cocaine properties by changing the BDNF expression in specific brain regions [72,73].

Family, twin, and adoption studies have shown that heritable factors play an outstanding role in determining individuals’ vulnerability to AUD. Many AUD-associated genetic variants have been identified by genome-wide association studies (GWASs). In genome-wide studies, methylation was found to be an important process in connection with alcohol abuse. In comparing alcoholics with their non-alcoholic siblings, the authors have found that several genes have altered methylation signatures, such as *ALDH1L2* (aldehyde dehydrogenase gene), *GABRP* (GABA receptor gene), and *GAD1* (glutamate decarboxylase gene), which are linked to the alcohol tolerance dopamine beta-hydroxylase gene (*DBH*) [74]. In their study, Bruckmann et al. have confirmed a genome-wide report of hypomethylation in the ganglioside-induced differentiation-associated protein 1 (*GDAP1*) gene and the association between the DNA methylation of this gene and the disease severity of 49 AUD patients [75]. The authors have also observed that the hypomethylation of *GDAP1* in patients was reversed during a short-term alcohol treatment program, and this may suggest that *GDAP1* DNA methylation could serve as a potential biomarker for treatment outcomes. Another study has shown a significant association of hypermethylation in the 3′-protein-phosphatase-1G gene (PPM1G) with alcohol use disorder, as well as two established AUD risk factors—adolescent escalation of alcohol intake and impulsivity [76]. The authors carried out a genome-wide analysis of the DNA methylation of 18 monozygotic twin pairs discordant in terms of alcohol use disorders and provided information on the association of the observed changes with brain mechanisms and behaviors that underline future problems associated with alcohol abuse. While genome-wide association studies (GWASs) have identified many AUD-related genetic variants, they only explain a small part of this puzzle. The genome-wide polygenic score (GPS) seems to be useful for identifying the risk of harmful and hazardous alcohol use [77], and because alleles do not change during an individual’s lifetime, GPS can be used to indicate an individual’s behavioral predispositions from birth. GPS based on a GWAS of alcohol-related behaviors has been shown to efficiently predict alcohol consumption. Using GPS based on the genome-wide association study and sequencing consortium of alcohol and nicotine use (GSCAN), with a cohort study of 3390 subjects, the authors have observed that the utility of GPS is limited in terms of the prediction of individual levels of alcohol use [77]. They have observed an increase in the predictive validity of a GPS for alcohol use from age 16 to 22 years by 5% for alcohol consumption, 90% for alcohol intake frequency, and 11% for hazardous drinking, with a generally small effect size, and they concluded that the clinical utility of GPS for alcohol use seems to be limited.

DNA methylation studies on AUD are still developing, as the role of DNA methylation in other diseases, such as cancer, is relatively easier to assess. There are also limited tissues to analyze in AUD, including mainly blood, saliva from living persons, and post-mortem brain. The potentiality of peripheral blood global DNA methylation as an AUD marker was analyzed by Bonsch and Kim [78,79]. Bonsch et al. examined the relationship among global methylation, plasma homocysteine, and AUD in a case control sample, which consisted of 90 AUD patients and 89 healthy controls. They found an increase in global methylation in AUD patients, who had higher levels of homocysteine. Kim et al. reported elevated methylation levels of the repetitive element, Alu, in peripheral blood DNAs in an AUD group, when comparing 135 AUD patients and 150 healthy controls. The DNA methylation of promoter regions of genes has also been examined. In the work of Hillemacher et al. [80], arginine-vasopressin (*AVP*) and atrial natriuretic peptide (*ANP*) promoter DNA methylation were compared. Their study was composed of 111 AUD subjects and 57 controls, and they observed a significant increase in *AVP* promoter DN methylation but also a decrease in the *ANP* promoter DNA methylation in AUD persons. An important element of alcohol dependence studies is the identification of links between epigenetic factors and addiction risk (e.g., the intensity of alcohol cravings and dealing with it or differences in the tendency to relapse) and the possible relationship with predictors of abstinence or alcohol consumption control. Interestingly and importantly in terms of addiction therapy, Lesh’s subtypes have described the causes of alcohol cravings; for example, in subtype I, the cause is alcohol consumption, stress in subtype II, depressed mood in subtype III, and in subtype IV, it is compulsive seeking [81]. The studies of Hillemacher et al. and Nieratschker et al. showed a significant negative correlation between the methylation of the dopamine transporter gene (*DAT*) and alcohol cravings [82,83]. The Polish study indicated a connection between the subtype I of alcohol dependence of Otto Michael Lesh and the trait of temperament, “novelty seeking”. Moreover, the “novelty seeking” trait is considered to be a predictor of alcohol consumption relapse and the worst prognostic indicator of abstinence. Neurobiological concepts of alcohol dependence describe addicted patients, with significant emphasis on the “novelty seeking” trait, as impulsive and disordered, and behaviors and emotions of this kind are conditioned by the dopaminergic system [84]. Moderate hyperhomocysteinemia is common in chronic heavy alcohol drinking. Due to the gradation of alcohol dependence and polyetiology of addiction (multigeneity), the relationship between the epigenetic changes (observed as DNA methylation within homocysteine-induced endoplasmic reticulum (ER) protein promoter (*Herp*)) and Lesh’s alcohol dependence typology was also noticed [85,86]. Additionally, other gene-specific investigations have observed changes in neuronal tract neuromodulators connected with alcohol cravings, such as proopiomelanocortin (*POMC*) and alpha-synuclein (*SNCA*) [87]. Studies have also highlighted a cluster of DNA methylation site alterations within the *POMC* promoter, which were correlated with alcohol cravings, both prior to (pre-exposure) and after alcohol consumption (post-exposure), in alcohol use disorders [88]. Analysis of the *SNCA* promoter in 84 AUD persons has shown hypermethylation in alcoholics in the acute exposure and post-exposure withdrawal phases, when comparing them with 93 controls [89]. Philibert et al. observed a meaningful association between the degree of alcohol dependence and the methylation status of monoamine oxidase A (*MAOA*) gene methylation in female patients (96 subjects) but not in men (95 subjects) [90]. Despite the many discrepancies, all these observations are promising and suggest the discovery of biomarkers in the future, indicating a strong need to expand research on the role of epigenetic mechanisms and, especially, DNA methylation in alcohol addiction, which, in the future, may serve as the basis for epigenetic control in these patients.

### 3.2. Methylation and Acetylation of Histones

Histone proteins are composed of a central globular domain and N-terminal tails, which are a subject of many chemical modifications. From the epigenetic point of view, the most important modifications include acetylation and methylation [54]. Histone acetylation is established by histone acetyltransferase (HAT) activity and leads to the addition of the acetyl group to lysine. Conversely, histone deacetylases (HDACs) remove the acetyl groups from the histone tails. Both of these groups of enzymes have been linked to psychiatric disorders and part of the addiction mechanism [91]. Studies on animal models of addiction have shown that many psychoactive substances may induce changes in histone modification in the central nervous system [91,92], and alcohol is one of them, as acute alcohol intoxication can decrease HDAC levels and histone acetylation in mouse amygdala [93]. One of the first studies in this area, which focused on epigenetic modulation due to the effect of alcohol, was carried out on rat hepatocytes treated in vitro with ethanol in a dose- and time-dependent manner. The authors observed an increase in the acetylation of the lysine K9 of histone H3 (H3K9ac) [94], the transcription activity of which plays an important role in apoptosis. Further studies have confirmed the role of the modification of HAT activity in addiction. In the nucleus accumbens (NAc), an increase in the global histone H3 and H4 acetylation was observed, and according to the authors, these regions possibly experience transcription activation at important and specific genomic locations that are relevant to AUD [95]. It has also been observed that binge-like exposure in adolescent rats induced HAT activity in the pre-frontal cortex and resulted in H3Kac and H4Kac in the promoters of genes, which are important in synaptic plasticity and transcriptional mechanisms [96]. The modulation in H3K9ac was also observed in rats after the acute administration of ethanol in vivo in the liver, lung, and spleen tissues [97]. Studies using HDAC inhibitors have shown a link between addiction and epigenetic modifications. The administration of HDAC inhibitors, such as 5-azacitidine (5-AzaC), decreases ethanol consumption and self-administration and inhibits behavioral responses due to ethanol consumption [98]. While all these findings are important, they are not universal, and of course, they are highly dependent on many changes, such as the protocol of administration during animal model experiments.

The methylation of histones is similarly regulated by two sets of enzymes: histone methyltransferase and demethylase, which are responsible for adding and removing the methyl group from amino acids. The methylation of histones is much more complicated, as it may occur at different sites—17 lysine and 7 arginine residues—and may occur as mono-, di-, or trimethylation [99]. Histone methylation plays an important role in regulating psychiatric disorders, including AUD, and many studies have been undertaken to explore this issue. For instance, it has been shown that the activation of H3K4me2 mark was increased alongside the histone acetylation at the promoters of transcription factors (TFs) in adolescent rats, after binge-like alcohol exposure [96], and acute EtOH exposure in mice significantly increased the levels of H3K4me3 in the cortex [100]. In spite of this, opposite effects have also been observed, and acute EtOH exposure in the amygdala of adult rats has resulted in a reduction in H3K4me3 at the promoters implicated in alcohol dependence [101]. In humans, a similar observation has been made. A study of the amygdala and frontal cortex of post-mortem tissues in alcoholics has shown that a globally increased H3K4me3 was observed [102].

### 3.3. Epigenetic Changes of BDNF

Approximately 20 years ago, Koob and colleagues proposed an “allostasis model” of alcohol addiction. According to this model, prolonged and excessive alcohol exposure may produce adaptive changes in brain function. These changes may lead to aberrations in the brain’s homeostatic system. Allostasis refers to integrative adaptive processes maintaining stability during changes, but this stability is not within a normal homeostatic range [103].

There are some important neurotransmitters, which are the most important players in this allostasis process, and many are part of the stress-response system [104]. Among them, one can find the corticotrophine-releasing factor (CRF), dynorphin (DYN) and its receptor (the κ opioid receptor), substance P, norepinephrine, and brain-derived neurotrophic factor (BDNF). BDNF is a neurotrophin that regulates neuronal growth, survival, and function during the development of the adult brain. This factor regulates synaptic transmission and plasticity and induces an increase in the cytosolic calcium content, which can affect the vesicle exocytosis of several neurotransmitters in the synaptic space [105]. It also modulates the cAMP-responsive element-binding (CREB) protein activity, activates the mitogen-activated protein kinase (MAPK) cascade, and influences gene expression, such as the activity-related cytoskeleton-associated (Arc) protein, which regulates synaptic plasticity [93,106]. Dysregulation of BDNF expression, most likely due to epigenetic changes, is observed in alcohol-dependent individuals [107]. Moreover, it has been shown that alcohol exposure can increase the phosphorylation of CREB, CREB-binding protein (CBP) levels, and the expression of BDNF and Arc in the specific parts of the brain of experimental rats [108]. These results underline that in amygdaloid circuitry deficit in CREB, signaling may lead to chromatin remodeling and a reduction in the BDNF and Arc expression, promoting excessive alcohol consumption and a heightened anxiety-like behavior. On the other hand, BDNF knockdown animals exhibited a depressive-like behavior and consumed higher amounts of alcohol [109]. In humans, the valine 66 to methionine (Val66Met) polymorphism within the BDNF sequence is associated with psychiatric disorders [110]. In addition, it was shown that mice carrying the Val68Met polymorphism (a mouse homolog of human Val66Met) are at a higher risk of developing uncontrolled and excessive alcohol consumption patterns [111]. These data support the hypothesis that deficiency in BDNF due to the Met68BDNF polymorphism in mice has an essential role in promoting alcohol consumption and suggest that this polymorphism plays a role in excessive and compulsive alcohol consumption. It is possible that “allostase” is a result of chronic alcohol consumption, and it can be maintained by an inappropriate diet, which is preferred by addicted individuals. Some studies suggest that diet modifications may have a great influence on neurotransmitter balance [112].

### 3.4. MicroRNAs

An important epigenetic process regulating gene expression also works through microRNA (miRNA). miRNAs are small, conserved noncoding RNA molecules consisting of 21–24 nucleotides that act at the post-transcriptional level to regulate the expression of their respective target messenger RNA (mRNA) and encoded proteins [113]. In the case of addiction to many substances, such as alcohol, methamphetamine, and nicotine, differences in the miRNA profile between substance users and a healthy control group have been observed [114,115,116]. It was shown that in animal models, the depletion of miRNA in central nervous system structures (due to the suppression of Drosha and Dicer enzymes) can alter neuronal growth and maturation [117,118]. A substantial number of studies have observed that specific microRNAs are modulated in alcohol abuse [119]. Selected members of the microRNA (miRNA) family are affected by alcohol, resulting in an abnormal miRNA profile in the liver and circulation in ALD [120]. In addition, there is increasing evidence that miRNAs responsible for inflammation regulation and cancer-promoting lipid metabolism are affected by excessive alcohol administration in mouse alcoholic liver disease (ALD) models. In addition, some studies have assessed the role of miRNAs in ALD and non-alcoholic fatty liver disease [121].

Interestingly, some studies have also indicated that moderate levels of alcohol consumption may have beneficial health effects and have shown that moderate voluntary alcohol consumption in Wistar rats affect many changes to the gene expression related to colonic inflammation and antioxidant enzymes. In the alcohol-treated animal group, a lower level of 8-oxo-deoxyguanosine was found, which strongly suggests a decrease in oxidative stress [122]. There was also a lower level of alanine aminotransferase and lactate dehydrogenase, as well as a decreased level of cyclooxygenase-2 gene expression, which is an inflammatory marker. The findings show that the alcohol-consumption group had an increased expression of glutathione-S-transferase-M1 and aldehyde dehydrogenase 2, indicating that moderate levels of alcohol consumption may provide beneficial effects in terms of reducing colorectal cancer (CRC) risk [122], which is consistent with the results of some human studies [123].

## 4. Nutrition

Environmental epigenetic effect factors include behavior, nutrition, and chemical and industrial pollutants. For example, bioactive food components may trigger life-protecting epigenetic modifications. Understanding the molecular effects of behaviors, nutrition, and pollutants has become relevant in the development of preventative strategies and personalized health programs [124]. “Environmental epigenetics” refers to how environmental exposure affects epigenetic changes. Nutrition is one the most important environmental epigenetic factors. Nutritional epigenetics is a quite recent subfield of epigenetics, so current knowledge on the precise effects of food components on epigenetics and their association with phenotypes is still elusive. As was observed, bioactive food components, specific nutrients, and dietary patterns may have highly beneficial effects and may also mitigate the negative impact of life behaviors, such as smoking, alcohol abuse, or exposure to certain chemicals.

### 4.1. Nutrition in Early Life

Nutrition in early life induces long-term changes in DNA methylation, which affect one’s health later in life. Nutrients may act directly by inhibiting epigenetic enzymes, such as DNMT, HDAC, and HAT, or may alter the accessibility of substrates important for these enzymes. This may result in the modification of the expression of genes critical for our health and longevity [125]. One brilliant example of the effect of early diets on epigenetics, with effects on the phenotype, is that of honeybees. Epigenetic changes in DNA methylation determined by a larval diet constitute the most important trigger. Larvae destined to become queens are fed exclusively with royal jelly, which contains epigenetically active ingredients that silence a key inhibiting “queen gene”. Royal jelly is a concentrated mixture of proteins, essential amino acids, unusual lipids, vitamins, and other less characterized compounds, and it is produced by the head glands of “nurse” workers. In honeybees (*Apis mellifera)*, genetically identical female larvae have been shown to change their development path depending on nutrition factors, such as royal jelly, and become queens, instead of workers, as required [126,127].

### 4.2. Epigenetic Effect of Diet on Health or Disease

Many studies have reported the epigenetic effect of diet on phenotypes and susceptibility to disease. The additional effect of nutrition can be considered when one focuses on vitamins. The epigenetic effects of 13 vitamins were recently described in terms of DNA methylation, histone modification, and ncRNA expression [128]. It is worth mentioning that sirtuin 1, a NAD+-dependent HDAC, the substrate specificity of which includes histone proteins, may be activated by dietary components, such as resveratrol. Sirtuin 1 mediates some dietary restriction effects by acting on DNA methylation [129].

### 4.3. Micronutrients in Epigenetic Modifications—The Vitamin B Group

The essential micronutrient folates, vitamin B6 and vitamin B12, are critically involved in homocysteine metabolism. This metabolism is linked to phenotypic changes through DNA methylation due to its role as a source of one-carbon for the synthesis of SAM (S-adenosyl methionine), which is crucial for DNA methylation [124] and nucleotide synthesis. Folic acid (B9) is an essential B vitamin, which plays a pivotal role in brain development as folate supplementation during early pregnancy, protecting against neural tube defects [130]. DNA and histone methylases are directly influenced by the accessibility of the methyl group derived from diet (choline/betaine, methyl folate, or methionine), which is needed for cytosine methylation in DNA or lysine in histones [131]. Folate and vitamin B12 are required to re-methylate homocysteine into methionine, while B6-dependent enzymes take part in converting homocysteine into cysteine. Other methyl donor nutrients, such as choline, can also impact DNA methylation status. Choline is a required nutrient, and foods such an eggs and meats contain more choline than plant sources. It has been observed that a low choline level in diets in pregnant rodents may result in changes in methylation. These changes are especially important, as a minimum level of maternal dietary choline is essential for normal brain development. Moreover, disruption in choline metabolism may affect DNA methylation by deleting the Bhmt gene [132]. In human maternal choline intake-modulated epigenetic placenta readings, women with a higher intake of choline had a higher placental promoter methylation in the corticotrophin-releasing hormone (CRH) [133]. In addition, babies born to women who consume more choline during pregnancy have been shown to have a better visuospatial memory at age 7 [134]. Equally important for methylation is the proper level of vitamins, as recently shown in the article by Tanwar et al. It was indicated that maternal vitamin B12 deficiency in rats alters fetal DNA methylation in metabolically important genes, and this impact may be reversed by B12 rehabilitation of mothers at conception [135]. This observation is promising in relation to therapy and prevention. A robust example of epigenome-modifying chemicals is bisphenol A (BPA), which is commonplace in the manufacture of numerous plastic products, including containers. It has been observed that the pups of BPA-fed adult mice were more likely to have an unhealthy phenotype, compared to those born to BPA-fed mothers supplemented with methyl-rich nutrients, such as folic acid and vitamin B12. As indicated, a good diet rich in fruits and vegetables and other high-quality foods may counteract the negative effects of chemical exposure. Methyl-donating nutrients act as co-substrates for methyl-group transfers. The pool of available methyl donors is a significant factor in DNA and histone methylation [136]. Alcohol has been shown to modulate vitamin B and folate synthesis in the body [137,138]. Therefore, it seems to be reasonable that alcohol may indirectly modulate DNA methylation through diet. Apart from this, alcohol metabolites, such as acetaldehyde, have been shown to modulate DNA methylation by inhibiting DNA methyl transferases [43].

### 4.4. Vitamin A

It was also shown that epigenetic memory, such as methylation, may be erased to produce naïve pluripotent stem cells, and vitamin A may reduce the DNA levels of 5-methylcytosine [139] by increasing ten-eleven translocation (TET) enzymes. Retinoid acid (RA), the active form of vitamin A, could induce differential gene expression through the DNA methylation of homeobox transcription factor A1 (HOXA1) and potential oncogene mucin 4 (MUC4) genes in two cancer breast lines [140]. The role of vitamin A is underlined by the function of a few ALDH isoforms (ALDH1A1, ALDH1A2, and ALDH1A3) in retinoic acid (RA), signaling by oxidizing retinal to retinoic acid (RA) [140]. According to Balmer and Blomhoff, there are over 500 genes, the expressions of which are up- or down-regulated by RA. Therefore, RA may modulate a variety of biological processes [141]. Vitamin E was also found to be an epigenetic factor due to the association between leukocyte methylation status and blood vitamin level in a Parkinson’s disease cohort [142]. It was shown that alcoholic liver function impairment leads to decreased serum vitamin A and vitamin E levels, although in relation to liver function impairment, a decrease in vitamin E seems to be more dependent on nutritional status and irregular eating habits. According to this study, both were related to data on brain atrophy and cerebellar shrinkage [143].

### 4.5. Vitamin D

Vitamin D is a steroid hormone that controls more than 1000 genes. Genes responsive to vitamin D may be categorized in a few groups consisting of genes involved in bone metabolism, anabolism, and resorption, mineral homeostasis, cell life, and immune-system modulation and metabolism. A deficiency of vitamin D is quite common due to restricted exposure to sunlight and/or a decreased dietary intake. Vitamin D binds to the receptor (VDR) that drives the expression of VDR-responsive genes. Furthermore, vitamin D interacts with epigenomes on many levels. Several studies have also shown the effect of vitamin D on the synthesis pathways of dopamine, serotonin, and a number of neurotrophic factors. In a study on the effect of vitamin D3 in children with attention-deficit/hyperactivity disorder (ADHD), it was found that the level of 25D3 and dopamine increased in the supplemented group, while the serum BDNF and serotonin levels did not change significantly [144]. Moreover, vitamin D is being reconsidered as a neuroactive steroid. The reported neuroprotective effects of vitamin D include the in vitro biosynthesis of neurotropic factors, the inhibition of nitic oxide synthase, and an increased level of brain glutathione. Vitamin D is a potent in vitro inducer of NGF mRNA expression in neural brain cells, and BDNF is a protein related to NFG, a central player in synaptic and cognitive plasticity [145]. In Pozzi et al. [146], the authors observed that after vitamin 25D3 supplementation, the level of NGF and BDNF in serum was lower than at the start of the trial, while the level of 25D3 had increased. They also observed a strong positive effect on memory and cognitive function, measured by the Wechsler Memory Scale. They conclude that the decreased level of NGF and BDNF after vitamin D supplementation may be connected, and supplementation plays a crucial role in the modulation of neurotrophic factors. Critical genes in the vitamin D signaling pathway, such as those coding for 25-hydroxylase (CYP2R1), 1α-hydroxylase (CYP27B1), and 24-hydroxylase (CYP24A1), as well as the vitamin D receptor (VDR), have a large CpG island in their promoter regions, which may be a potential methylation site. Additionally, VDR protein affects coactivators and corepressor proteins, which are in contact with HATs, HDACs, HMTs, and chromatin remodelers. There is also some evidence that certain VDR ligands have DNA-demethylating effects [147]. The epigenetic effects of vitamin D are connected with histone acetylation. For example, it was shown that the VDR/RXR dimer interacts with HATs to induce transcriptional activation [148]. Some authors have also proposed that vitamin D can alter DNA methylation in the promotion of certain genes. Tapp and colleagues showed the negative association between the serum level of 25D3 and CGI methylation in the adenomatous polyposis coli (APC) promoter region [149]. They also suggested that, in healthy people, the age-related CGI-methylation of human rectal mucosa was influenced by, among other things, the vitamin D status. In non-malignant and malignant prostate epithelial cells, after treatment with 1,25-D3, clear changes were observed in the site-specific methylation of the p21 promoter in a cell line-specific manner [150]. The precise mechanism of vitamin D as an epigenetic modifier needs further investigation.

### 4.6. Vitamin C

The epigenetic role of vitamin C has generally been proven. Vitamin C plays a pivotal role in remodeling epigenomes by enhancing the catalytic activity of Jumonji-C domain-containing histone demethylases (JHDMs) and the ten-eleven-translocation proteins (TETs). The ability of vitamin C to potentiate the activity of histone and DNA demethylating enzymes also has clinical applications in cancer treatment [139,151]. A variety of dietary factors are potential HDAC and HAT modulators. Among these, sulforaphane, found in broccoli sprouts, or diallyl disulfide in garlic have been shown to act as HDAC inhibitors [125]. All these examples have emphasized the role of diet and fresh air exercise in relation to the development of disorders, such as addiction, and Table 3 summarizes how individuals’ diets may influence epigenomes.

### 4.7. Nutrition and Cognitive Functions

It remains unclear why some epigenomes are established early, whereas others are modifiable in later life. It is well known that perinatal environmental conditions are very important and exert lasting effects on the brain function and structural development, as well as on the susceptibility to abuse and psychopathology later in mature life [156]. Nutrition is one of the most important elements of the parent–child relationship, which may affect offspring’s brain development and function. There is strong evidence suggesting a direct association between early-life stress (ELS) and the incidence of psychiatric disorders and cognitive impairment [157,158]. The quality of early nutrition has major effects on adult cognitive function. Feeding behaviors and metabolism are closely regulated by the neuroendocrine mechanism, which is affected by stressful events, and malnutrition also affects the stress system. The programming of the human brain is a very complex process, in which nutrition and stress play crucial roles. There is growing evidence that ELS and EL nutrition affect the hippocampal structure, plasticity, and function. It is common knowledge that the hippocampus is particularly sensitive to the EL environment due to its postnatal development between the last trimester of gestation and 16 years of age, and it is rich in stress-hormone receptors [159]. Proper brain development requires an adequate supply of energy and micro and macro nutrients. Even minor dietary insufficiencies may have a big impact, especially during critical stages of development. For example, children subjected to bad perinatal nutrition exhibit cognitive deficits and an increased risk of psychopathologies in adulthood [160,161]. While many nutrients are essential for neuronal growth and brain development, during the perinatal period, the intake of zinc, selenium, iron, folate, iodine, vitamin A, vitamin B6 and B12, long-chain-polyunsaturated fatty acids, choline, and proteins is of particular importance. There is some evidence that perinatal manipulation of nutritional status may induce an alteration in hippocampal neurogenesis and other structural changes in animals [162]. Moreover, vitamin B6 and B12 deficiencies during gestation and lactation persistently impair hippocampal structure and function, and protein malnutrition may result in a reduced neuronal DNA and RNA content, as well as altered fatty acid profiles, which may ultimately lead to serious changes in neuronal function, the number of synapses, and/or dendritic arborization [163,164].

It is well known that maternal care, stimulation, and nutrient availability are the most important factors in early development, and stress hormones and neuropeptides are particular components that should be considered in combination, as they mediate the long-lasting effects of early life experiences. One of the most important responses of the human organism to stress stimuli is the HPA-axis. Food intake and HPA-axis activity are closely linked to neuronal pathways that react to and integrate nutritional and stressful stimuli. The importance of this connection is confirmed in some studies, which show that basal HPA-axis activity and stress responsiveness are altered in genetically obese rats and in rodents fed a high-fat diet and subjected to perinatal food restriction [165,166]. The HPA-axis is sensitive to modulation by metabolic signals, including leptin, insulin, glucose, and ghrelin [167,168]. As was shown, maternal separation reduces plasma glucose and leptin and increases ghrelin levels in offspring [169]. All this suggests that metabolic signals are an important element of the HPA-axis response.

Patients with an alcohol use disorder often eat abnormally and irregularly. They experience nutrient deficit symptoms, alcohol withdrawal syndrome (vomiting, diarrhea, and sweating), and disturbed bacterial intestinal flora and nutrient absorption systems, resulting in vitamin, protein, and electrolyte deficiencies [170,171,172], which may lead to health damage and also the initiation of epigenetic processes [173].

### 4.8. Cannabinoids

Many studies have analyzed the influencing substances in food that may affect the nervous system and appetite reactions. These substances modulate the nervous system and nutritional behavior. Researchers have emphasized the role of the endogenous cannabinoid neuronal system in the regulation of food intake [174]. This system includes receptors in areas of the central nervous system, such as the lateral hypothalamus, arcuate nucleus (nucleus caudatus), and paraventricular nucleus (nucleus paraventricularis), and in the reward system, e.g., experiences of hunger modulated by hormones, such as leptin, orexin, and endogenous opioids [175,176,177,178,179]. Cannabinoid receptors, CB1 and CB2, are located in two metabolic long- and short-term systems of nutrition regulation connected with the repletion of the digestive system [180]. Cannabinoids intensify the rewarding potential of food intake. Animal model surveys have proved that the application of the receptor CB1 antagonist (Rimonabant, SR141716A) decreased the reward potential of eating sweets and alcohol consumption [181]. Interestingly, it has been proved that not only do human or animal organisms include cannabinoids but that these substances are also components of food (exogenous cannabinoids, e.g., cacao, chocolate, and milk) [182,183,184]. This leads to the conclusion that food may include additional biochemical substances that may induce specific physiological reactions and behavior. It seems to be also possible that these substances may indirectly increase the risk of alcohol consumption by eliciting the desire to use alcohol or that, on the contrary, these substances may be protection factors supporting abstinence. Studies have also indicated that DNA methylation may occur due to the use of psychoactive substances, e.g., alcohol and exogenous cannabinoids [177]. Researchers have emphasized interactions between ethanol and the cannabinoid system. Moreover, recent studies have shown that epigenetic changes that occur after alcohol consumption, together with cannabinoids, may act synergistically and lead to DNA methylation or histone modification. This in turn may lead to a modulation of apoptosis and synaptic plasticity [185]. Some reports reveal the ability of cannabinoids to modify the neuronal and immune system via histone modification, such as H3 lysine methylations or the alteration of DNA methylation [94].

## 5. Conclusions

The role of epigenetic modification, nutrition, and addiction, such as alcohol abuse, is an emerging and promising field of research. It seems to be highly reasonable to look for new connections between them, especially nowadays, when nutrigenomics and nutrigenetics are becoming important elements in disease and addiction therapies. Changes in human diet may be the easiest first stage of therapy or may fulfil a protective role in specific disorders, such as addiction. In light of all these observations, it is important to address whether the knowledge resulting from studies of the influence of epigenetic factors (e.g., the environment and alcohol) help to initiate preventive action, leading to a modification of patients’ environment, therapy, and diet.

## Figures and Tables

**Table 1:** Nomenclatures for human class I ADH variants ^a^.

Allele	Former Allele Nomenclature	Protein
ADH1A	ADH1	ADH1A
ADH1B*1	ADH2*1	ADH1B1
ADH1B*2	ADH2*2	ADH1B2
ADH1B*3	ADH2*3	ADH1B3
ADH1C*1	ADH3*1	ADH1C1
ADH1C*2	ADH3*2	ADH1C2

^a^ Based on Duester et al. and Quertemont [3,9].

**Table 2:** Human ALDH polymorphisms ^b^.

Major Substrate	Gene
Retinal	*ALDH1A1*
Aliphatic aldehyde, retinal	*ALDH1A6*
Retinal	*ALDH1A7*
Propionaldehyde	*ALDH1B1*
Folate	*ALDH1L1*
Acetaldehyde	*ALDH2*
Fatty and aromatic aldehyde	*ALDH3A1*
Fatty and aromatic aldehyde	*ALDH3A2*
Aliphatic and aromatic aldehyde	*ALDH3B1*
Glutamate γ-semialdehyde	*ALDH4A1*
Succinic semialdehyde	*ALDH5A1*
Methylmalonate semialdehyde	*ALDH6A1*
Amine aldehyde	*ALDH9A1*

^b^ Based on Vasiliou et al. and Quertemont [3,11].

**Table 3:** Dietary components and their epigenetic role ^c^.

Food Items	Nutrient	Epigenetic Role
Fish, peppers, spinach, sesame seeds, brazil nuts, meats, eggs, parmesan, flax seeds, pumpkin seeds and sunflower seeds, brussels sprouts, broccoli, spinach, peas, beans	Methionine	Used in SAM synthesis
Lettuce, spinach, broccoli, cabbage, cauliflower, brussels sprouts, asparagus, broad beans, green peas, beets, tomatoes, sunflower seeds, baker’s yeast, liver, nuts, whole grain bread, eggs, cheese	Folic Acid	Remethylation of homocysteine to methionine
Meats, whole grain products, nuts, broccoli, potatoes, wheat germ, baker’s yeast, soybeans, bananas, dairy products, fish, eggs	Vitamin B6	Cystathionine formation from homocysteine
Milk, liver, shellfish, meat, fish, eggs, cheese, cold cuts, baker’s yeast	Vitamin B12	Cofactor for methionine synthase
Sugar beets, shellfish, spinach, wheat	Betaine	Cofactor for betaine-homocysteine methyltransferase, homocysteine methylation to methionine
Cocked beef, chicken, veal, turkey, soy, liver, egg yolks	Choline	The source of betaine
Red wine, grapes	Resveratrol	Modulation of DNA methyltransferase (DNMT), HDAC and lysine-specific demethylase-1 (LSD1) activity
Garlic	Diallyl sulfide (DADS)	Inhibitor of HDAC
Vegetable oils, nuts, pumpkin seeds, sunflower seeds, sesame, almonds, wheat germ, avocado, lard, margarine, eggs, halibut, butter, oatmeal, rye bread	Vitamin E	Induction of MLH1 and DNMT1 gene expression
Parsley leaves, pepper, brussels sprouts, kohlrabi, broccoli, cabbage, cauliflower, spinach, rosehips, black currants, strawberries, kiwi, grapefruits, lemons, oranges	Vitamin C	Cofactor for TET and Alkb proteins and DNA demethylation
		Cofactor for Jumonji proteins and histone demethylation
Carrots, kale, parsley leaves, spinach, chard, sorrel, chives, pumpkin, dried apricots, garden fennel	β-carotene, Vitamin A	Increase in DNA demethylation in a TET-dependent manner
Liver, sweet potatoes, carrots		Increase in H3K9 and H3K14 acetylation

^c^ According to Tiffon et al. [124], Hore et al. [139], Cimmino et al. [151], Zappe et al. [152], Feng et al. [153], Bertolo et al. [154], and Fernandes et al. [155].
